# Electrophysiological stimulation is associated with improved lower-extremity venous hemodynamics and postoperative recovery following laparoscopic nephrectomy

**DOI:** 10.3389/fsurg.2026.1854870

**Published:** 2026-09-03

**Authors:** Yuwen He, Li'e Lin, Ningning Tian, Jiamei Chen, Yanping Wang, Sijing Huang, Yongnan Li, Li Lan, Yun Xie, Zihao Feng, Peiwei Wang, Yingwen Zhu, Xiaoping Huang

**Affiliations:** The First Affiliated Hospital, Sun Yat-sen University, Guangzhou, Guangdong, China

**Keywords:** electrophysiological stimulation, laparoscopic nephrectomy, postoperative recovery, thromboprophylaxis, venous hemodynamics, venous thromboembolism

## Abstract

**Background:**

Venous thromboembolism (VTE) is a common and potentially serious complication following laparoscopic nephrectomy. Conventional preventive measures are limited by bleeding risk and poor compliance. Electrophysiological stimulation therapy has emerged as a non-invasive approach to improve venous circulation, but its perioperative efficacy remains unclear.

**Methods:**

This prospective, non-randomized controlled study included 108 patients undergoing laparoscopic nephrectomy. Patients were allocated to a control group (*n* = 57) receiving routine VTE prophylaxis or an observation group (*n* = 51) receiving additional electrophysiological stimulation therapy. Venous blood flow velocity, Caprini score, D-dimer levels, and clinical recovery indicators were assessed preoperatively (T0) and before the first ambulation (T1). Change values (Δ = T1−T0) were analyzed. Multivariate regression and correlation analyses were performed.

**Results:**

Compared with the control group, the observation group showed significantly higher postoperative venous blood flow velocities in the popliteal, posterior tibial, and fibular veins (all *P* < 0.05). Δ analysis demonstrated greater improvements in venous hemodynamics (all *P* < 0.05). Hospital stay (6.86 ± 2.39 vs. 7.98 ± 2.44 days, *P* = 0.018) and bed rest duration were significantly reduced. Regression analysis identified electrophysiological therapy (*β* = −0.98, *P* = 0.018) and improved venous flow (*β* = −0.42, *P* = 0.006) as independent predictors of shorter hospitalization. Venous flow improvement was moderately negatively correlated with hospital stay (r = −0.36 to −0.41).

**Conclusion:**

Electrophysiological stimulation therapy was associated with improved venous hemodynamics and postoperative recovery following laparoscopic nephrectomy and may serve as a useful adjunct to routine perioperative thromboprophylaxis.

## Introduction

Venous thromboembolism (VTE) refers to the formation of thrombi within the venous system that obstruct blood flow, encompassing deep vein thrombosis (DVT) and pulmonary embolism (PE). It is currently recognized as the third leading cause of cardiovascular-related mortality worldwide and represents a significant burden on healthcare systems (1). Patients undergoing laparoscopic nephrectomy are at increased risk of VTE due to multiple perioperative factors. The establishment of pneumoperitoneum using carbon dioxide can lead to reduced venous return and venous stasis, while surgical stress contributes to a hypercoagulable state, collectively promoting thrombus formation (2). Previous studies have reported that the incidence of VTE in patients undergoing laparoscopic urological surgery is approximately 7.6% in domestic populations (2), whereas the incidence following laparoscopic radical nephrectomy has been reported to be around 1% in international studies. However, the precise incidence in patients undergoing laparoscopic nephrectomy in China remains unclear. Importantly, the occurrence of VTE is associated with prolonged hospitalization, reduced quality of life, and increased mortality, underscoring the need for effective preventive strategies (3). VTE is largely preventable, and timely implementation of appropriate prophylactic measures is essential, particularly in high-risk patients (4). Current preventive strategies include basic measures, mechanical methods, and pharmacological interventions. However, pharmacological prophylaxis may increase the risk of postoperative bleeding, while mechanical methods such as graduated compression stockings and intermittent pneumatic compression devices often require prolonged use and may be associated with poor comfort and reduced patient compliance, potentially limiting their effectiveness (5, 6). Consequently, there is increasing interest in adjunctive non-pharmacological strategies that may enhance venous return without increasing bleeding risk.

Electrophysiological stimulation techniques, which integrate principles of traditional Chinese medicine with modern electrophysiology, have emerged as a potential alternative approach for improving lower-extremity venous circulation during the perioperative period (7). By delivering low-frequency electrical stimulation to the lower limbs, these techniques induce rhythmic muscle contractions that enhance venous return and improve local blood circulation. Compared with conventional mechanical methods, electrophysiological therapy is non-invasive, well tolerated, and may offer improved patient compliance. Previous studies have demonstrated favorable effects of electrical stimulation on lower-extremity blood flow and venous hemodynamics in various surgical and non-surgical populations (8–13). However, evidence regarding its application in patients undergoing laparoscopic nephrectomy remains limited, and its potential influence on postoperative venous hemodynamics and recovery has not been adequately investigated.

Therefore, the present prospective study aimed to evaluate the effects of adjunctive electrophysiological stimulation on lower-extremity venous hemodynamics and postoperative recovery in patients undergoing laparoscopic nephrectomy receiving routine thromboprophylaxis. We hypothesized that electrophysiological stimulation would improve postoperative venous blood flow and facilitate recovery without increasing treatment-related adverse events.

## Materials and methods

### Ethics statement

This study was conducted in accordance with the ethical principles of the Declaration of Helsinki. The study protocol was reviewed and approved by the Ethics Committee of the First Affiliated Hospital of Sun Yat-sen University (Approval No. 2021-005). Written informed consent was obtained from all participants or their legal guardians prior to enrollment. All study procedures were performed in accordance with institutional ethical standards and Good Clinical Practice (GCP) principles, where applicable.

### Study design and participants

This study was designed as a single-center, prospective, non-randomized controlled clinical study to evaluate the effect of adjunctive electrophysiological stimulation on postoperative lower-extremity venous hemodynamics in patients undergoing laparoscopic nephrectomy.

**Figure 1:**
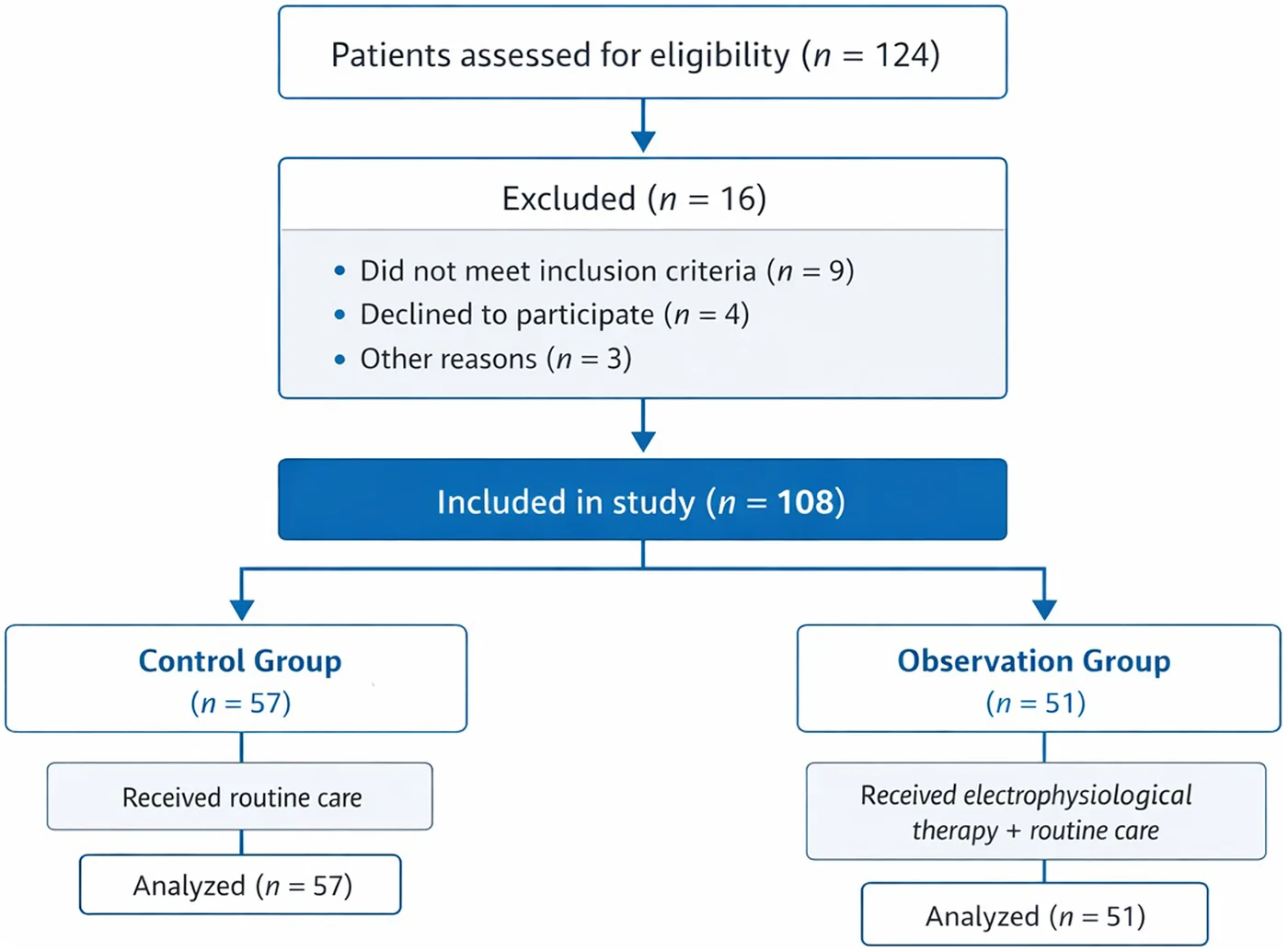
Flow diagram of participant enrollment and study allocation. A total of 124 patients undergoing laparoscopic nephrectomy were screened for eligibility. After application of the inclusion and exclusion criteria, 108 eligible patients were enrolled and allocated to the control group (*n* = 57) or the observation group (*n* = 51). All enrolled participants completed the study and were included in the final analysis.

A total of 124 consecutive patients who underwent laparoscopic nephrectomy at the Department of Urology, First Affiliated Hospital of Sun Yat-sen University between March 2025 and October 2025 were screened for eligibility. Following application of the predefined inclusion and exclusion criteria, 16 patients were excluded, including nine who did not meet the eligibility criteria, four who declined participation, and three for other reasons. Consequently, 108 eligible patients were consecutively enrolled and included in the final analysis. Because electrophysiological stimulation was sequentially introduced into routine perioperative management during the study period, participants were allocated according to the time of admission rather than by randomization. Patients admitted between March and May 2025 were assigned to the control group (*n* = 57), whereas those admitted between May and October 2025 were assigned to the observation group (*n* = 51). This pragmatic allocation strategy reflected routine clinical practice and ensured that all eligible patients received a consistent perioperative management protocol within each study period. The study flow is presented in Figure 1. Although time-based allocation may introduce temporal confounding and selection bias, the perioperative management protocol, surgical indications, and postoperative care pathways remained unchanged throughout the study period. Furthermore, baseline demographic and clinical characteristics were compared between groups, and multivariable regression analyses adjusting for clinically relevant covariates were performed to reduce the potential influence of residual confounding. Nevertheless, the possibility of unmeasured confounding cannot be completely excluded because of the non-randomized study design.

### Inclusion and exclusion criteria

Eligible participants were adult patients aged ≥18 years who underwent laparoscopic nephrectomy and had normal visual, auditory, speech, and cognitive functions, allowing adequate cooperation with treatment and assessment procedures. Written informed consent was obtained from all patients or their legal guardians prior to enrollment.

Patients were excluded if they had contraindications to electrophysiological therapy, including implanted electronic devices such as pacemakers, or local conditions at the electrode placement sites, such as skin infection, ulceration, or sensory abnormalities. Patients with conditions that could affect the occurrence or assessment of venous thromboembolism, including preoperative coagulation disorders, a history of VTE, or neuromuscular diseases impairing lower limb movement or responsiveness to electrical stimulation, were also excluded. Additional exclusion criteria included severe systemic diseases, including clinically significant cardiovascular, hepatic, or renal dysfunction, acute infections, pregnancy or lactation, severe mental disorders, inability to cooperate with study procedures, or withdrawal from the study.

### Interventions

Patients in the control group received routine nursing care for VTE prevention. This included preoperative risk assessment using the Caprini score, standardized health education, postoperative use of antithrombotic compression stockings, guidance on early mobilization and ankle pump exercises, and encouragement of adequate hydration and bowel function recovery when clinically appropriate.

Routine thromboprophylaxis was administered in accordance with institutional perioperative protocols and individualized based on each patient's Caprini risk assessment and bleeding risk. Patients at low risk of VTE received mechanical thromboprophylaxis, including graduated compression stockings, early mobilization, and ankle pump exercises. Patients with moderate or high VTE risk, in the absence of contraindications, additionally received pharmacological thromboprophylaxis with low-molecular-weight heparin (LMWH) according to institutional practice. The thromboprophylaxis strategy was identical in both groups throughout the study period; the only difference between groups was the addition of electrophysiological stimulation in the observation group. In addition to routine care, patients in the observation group received electrophysiological stimulation therapy administered by nurses who had undergone standardized training. The intervention was initiated on the first postoperative day using a low-frequency neuromuscular electrical stimulator (BioStimble, Foshan Shanshan Datang Medical Technology Co., Ltd., China; Yue Medical Device Registration No. 20182260716). Two pairs of electrode pads were placed bilaterally at the midpoint of the inguinal ligament corresponding to the femoral artery pulse and at the dorsalis pedis artery pulse sites. Electrical stimulation was delivered at a frequency of 8 Hz and a pulse width of 260 *μ*s. Each session lasted 30 min and was performed twice daily. The stimulation intensity was gradually increased from 0 mA until a perceptible but painless muscle contraction was achieved. The stimulation intensity was individualized according to each participant's tolerance and adjusted to produce visible rhythmic muscle contractions without causing discomfort or pain. Patients were continuously monitored by trained nursing staff throughout each treatment session to ensure treatment tolerance and safety. The intervention was continued until the patient achieved first postoperative ambulation. Treatment adherence was excellent, with all participants completing the prescribed electrophysiological stimulation protocol. One participant reported mild transient discomfort at the electrode placement site; however, the symptom resolved spontaneously without treatment interruption or discontinuation. No serious intervention-related adverse events occurred during the study period.

### Outcome measures

All outcome measurements were obtained at two predefined time points: preoperatively (T0) and at T1, which was scheduled at approximately 24 h after surgery and performed before the first postoperative ambulation according to the predefined study protocol. The timing of the T1 measurements was standardized for all participants and did not differ between the intervention and control groups. Although patients in the intervention group achieved earlier postoperative ambulation thereafter, this did not influence the scheduled timing of outcome assessment, thereby minimizing potential measurement bias related to differences in ambulation timing.

The primary outcome was the change in lower-extremity venous hemodynamics following electrophysiological stimulation, assessed by venous blood flow velocity. Venous blood flow velocity was assessed using color Doppler ultrasonography (ACUSON Sequoia, Siemens Healthineers, Erlangen, Germany) by experienced sonographers who were blinded to group allocation under standardized resting conditions. Patients were examined in the supine position after resting for at least 10 min of rest using a 5–12 MHz linear-array transducer. Blood flow velocity was measured during quiet respiration with the Doppler insonation angle maintained at ≤60°. Three consecutive measurements were obtained at each anatomical site, and the mean value was used for statistical analysis. Measurements were obtained bilaterally from the femoral vein, popliteal vein, posterior tibial vein, and fibular vein, and the average values were recorded for analysis.

Secondary outcomes included thrombotic risk assessment using the Caprini scoring system, which was evaluated at both time points. Plasma D-dimer levels were measured using standard laboratory assays to reflect coagulation and fibrinolytic activity. Limb circumference was assessed to evaluate postoperative swelling, with thigh circumference measured at 15 cm above the superior border of the patella and calf circumference measured at 10 cm below the inferior border of the patella. Clinical recovery indicators included length of hospital stay, defined as the duration from surgery to discharge; duration of bed rest, defined as the time from surgery to first ambulation; and time to first postoperative passage of flatus, reflecting recovery of gastrointestinal function. These secondary outcomes were included to explore the potential association between improvements in venous hemodynamics and postoperative recovery and were not intended to establish reductions in clinical VTE incidence. To further evaluate the magnitude of treatment effects, change values (Δ) were calculated for continuous variables as the difference between postoperative and preoperative measurements (Δ = T1 − T0).

### Statistical analysis

A formal *a priori* sample size calculation was not performed. This prospective study was conducted as an exploratory investigation, and all eligible patients who met the inclusion criteria during the study period were consecutively enrolled. A *post hoc* power analysis was performed using G*Power (version 3.1.9.7; Heinrich Heine University Düsseldorf, Düsseldorf, Germany) based on the primary outcome. Statistical analyses were performed using SPSS version 26.0 (IBM Corp., Armonk, NY, USA). Data normality was assessed using the Shapiro–Wilk test before statistical analyses. Continuous variables with normal distribution were expressed as mean ± standard deviation and compared between groups using independent-samples *t*-tests. Non-normally distributed data were expressed as median (interquartile range) and analyzed using the Mann–Whitney *U* test. Categorical variables were presented as frequencies and percentages and compared using the *χ*^2^ test. Changes in outcome variables were calculated as Δ values (T1−T0), and intergroup comparisons of Δ values were performed using appropriate parametric or non-parametric tests. Correlation analysis was conducted using Pearson or Spearman correlation coefficients to assess the relationships between changes in venous blood flow velocity and postoperative clinical outcomes. Multivariate linear regression analysis was performed to identify independent factors associated with hospital stay, with clinically relevant variables and those with *P* < 0.10 in univariate analyses included in the model. Model assumptions, including multicollinearity, residual normality, linearity, and homoscedasticity, were evaluated before interpretation of the regression results. Regression coefficients (*β*) and 95% confidence intervals (CIs) were reported. Because this was an exploratory study, no formal adjustment for multiple comparisons (e.g., Bonferroni or Holm correction) was performed. The primary outcome was prespecified, whereas analyses of secondary outcomes were considered exploratory and should therefore be interpreted with appropriate caution. All statistical tests were two-sided, and a *P* value < 0.05 was considered statistically significant.

## Results

### Baseline characteristics

A total of 108 patients were included in the final analysis, comprising 57 patients in the control group and 51 patients in the observation group**.** No statistically significant differences were observed between the two groups with respect to baseline demographic or perioperative characteristics, including age, sex distribution, body mass index (BMI), operation time, operation method, and operation site (all ***P*** > 0.05), indicating good baseline comparability (Table 1).

**Table 1:** Baseline demographic and perioperative characteristics of the study population.

Variable	Control Group (*n* = 57)	Observation Group (*n* = 51)	*t*/*χ*^2^	*P*-value
Age (years)	55.19 ± 12.99	55.12 ± 12.94	0.03	0.976
Male/Female, *n*	34/23	25/26	1.22	0.272
BMI (kg/m^2^)	23.61 ± 3.17	23.96 ± 3.41	−0.54	0.587
Operation time (min)	154.09 ± 59.73	143.75 ± 60.55	0.89	0.374
Operation method, *n* (%)	Radical nephrectomy: 35 (61.4) Partial nephrectomy: 22 (38.6)	Radical nephrectomy: 31 (60.8) Partial nephrectomy: 20 (39.2)	0.004	0.952
Operation site, *n* (%)	Left: 30 (52.6) Right: 27 (47.4)	Left: 24 (47.1) Right: 27 (52.9)	0.32	0.574

Values are presented as mean ± standard deviation (SD) or number (%), as appropriate. Continuous variables were compared using the independent-samples *t*-test, whereas categorical variables were compared using the χ2 test.

### Comparison of clinical outcomes

As shown in Table 2, the observation group was associated with significantly improved postoperative recovery compared with the control group. Specifically, the length of hospital stay was significantly shorter in the observation group than in the control group (6.86 ± 2.39 vs. 7.98 ± 2.44 days, *P* = 0.018). Similarly, the duration of bed rest was significantly reduced in the observation group (2954.67 ± 797.47 vs. 3434.26 ± 1278.16 min, *P* = 0.023). No statistically significant differences were observed between the two groups in time to first postoperative passage of flatus, Caprini score at T0 or T1, or D-dimer levels at T0 or T1 (all *P* > 0.05). No clinically confirmed cases of deep vein thrombosis or pulmonary embolism were observed in either group during hospitalization. In addition, all participants assigned to electrophysiological stimulation completed the planned intervention protocol. One participant reported mild transient discomfort at the electrode placement site, which resolved spontaneously without treatment interruption or discontinuation. No serious intervention-related adverse events occurred during the study period.

**Table 2:** Comparison of clinical outcomes between the two groups.

Variable	Control Group (*n* = 57)	Observation Group (*n* = 51)	*t*	*P*-value
Length of hospital stay (days)	7.98 ± 2.44	6.86 ± 2.39	2.40	0.018
Duration of bed rest (min)	3434.26 ± 1278.16	2954.67 ± 797.47	2.31	0.023
Time to first postoperative flatus (days)	2.02 ± 0.92	1.80 ± 0.63	1.39	0.166
Caprini score (T0)	2.53 ± 1.26	2.24 ± 1.48	1.11	0.271
Caprini score (T1)	4.11 ± 2.02	4.69 ± 1.93	−1.53	0.130
D-dimer (mg/L, T0)	0.64 ± 0.64	0.62 ± 0.96	0.15	0.885
D-dimer (mg/L, T1)	3.21 ± 2.78	2.75 ± 1.55	1.05	0.295

Values are presented as mean ± SD. Comparisons between groups were performed using the independent-samples *t*-test.

### Comparison of limb circumference

The circumferential measurements of the thigh and calf at both T0 and T1 showed no statistically significant differences between the two groups (all *P* > 0.05), indicating that electrophysiological therapy did not significantly affect postoperative limb swelling (Table 3).

**Table 3:** Comparison of lower-extremity circumference measurements between groups.

Assessment	Variable	Control Group (*n* = 57) Median (IQR)	Observation Group (*n* = 51) Median (IQR)	*Z*	*P*-value
T0	Left thigh (cm)	45.5 (44.25–48.00)	46.5 (43.00–49.00)	1.585	0.418
	Right thigh (cm)	46.0 (44.25–48.00)	47.0 (43.50–49.00)	1.603	0.359
	Left calf (cm)	31.5 (29.00–34.00)	32.5 (30.50–35.00)	1.743	0.075
	Right calf (cm)	32.0 (29.00–33.75)	32.5 (30.70–35.00)	1.717	0.105
T1	Left thigh (cm)	45.5 (43.25–47.25)	46.0 (42.50–49.00)	1.576	0.450
	Right thigh (cm)	45.5 (43.25–48.00)	46.0 (43.00–49.00)	1.590	0.402
	Left calf (cm)	32.0 (29.00–34.25)	32.5 (30.00–35.50)	1.747	0.071
	Right calf (cm)	32.0 (29.00–34.00)	32.6 (31.00–36.00)	1.743	0.074

Values are presented as median (interquartile range [IQR]). Comparisons between groups were performed using the Mann–Whitney *U* test.

### Comparison of venous blood flow velocity

There were no statistically significant differences in baseline venous blood flow velocity between the two groups at T0 (all *P* > 0.05), confirming comparability prior to intervention (Table 4). After intervention (T1), the observation group was associated with significantly higher blood flow velocities in multiple lower-extremity veins compared with the control group. Significant differences were observed in the popliteal vein, posterior tibial vein, and fibular vein on both sides (all *P* < 0.05). In contrast, no statistically significant differences were found in femoral vein blood flow velocity between the two groups (all *P* > 0.05) (Table 4).

**Table 4:** Comparison of lower-extremity venous blood flow velocity between groups.

Assessment	Venous Blood Flow Velocity (cm/s)	Control Group (*n* = 57)	Observation Group (*n* = 51)	*t*	*P*-value
Preoperative (T0)	Left femoral vein	5.91 ± 1.57	5.99 ± 1.62	−0.28	0.783
	Right femoral vein	6.05 ± 1.63	6.25 ± 1.64	−0.62	0.536
	Left popliteal vein	4.21 ± 1.16	4.31 ± 1.28	−0.41	0.686
	Right popliteal vein	4.37 ± 1.17	4.38 ± 1.23	−0.03	0.976
	Left posterior tibial vein	2.71 ± 0.66	2.81 ± 0.70	−0.72	0.471
	Right posterior tibial vein	2.69 ± 0.70	2.82 ± 0.75	−0.89	0.375
	Left fibular vein	2.73 ± 0.74	2.64 ± 0.75	0.59	0.556
	Right fibular vein	2.72 ± 0.73	2.69 ± 0.75	0.19	0.848
Postoperative (T1)	Left femoral vein	6.13 ± 1.58	6.59 ± 1.84	−1.38	0.171
	Right femoral vein	6.12 ± 1.59	6.68 ± 1.81	−1.72	0.089
	Left popliteal vein	4.28 ± 1.11	4.98 ± 1.33	−2.98	**0** **.** **004**
	Right popliteal vein	4.38 ± 1.07	5.03 ± 1.30	−2.84	**0** **.** **005**
	Left posterior tibial vein	2.95 ± 0.65	3.24 ± 0.79	−2.08	**0** **.** **040**
	Right posterior tibial vein	2.90 ± 0.65	3.32 ± 0.89	−2.86	**0** **.** **005**
	Left fibular vein	2.76 ± 0.67	3.31 ± 0.92	−3.61	**<0** **.** **001**
	Right fibular vein	2.80 ± 0.66	3.24 ± 0.86	−3.01	**0** **.** **003**

Values are presented as mean ± standard deviation (SD). Between-group comparisons were performed using the independent-samples *t*-test. Bold indicates statistically significant differences (P < 0.05).

### Comparison of changes (Δ values)

To further assess the magnitude of intervention effects, changes in outcome variables (Δ = T1 − T0) were analyzed (Table 5). The observation group showed significantly greater increases in venous blood flow velocity compared with the control group, particularly in the popliteal veins (left: 0.67 ± 0.58 vs. 0.07 ± 0.45; right: 0.65 ± 0.61 vs. 0.01 ± 0.48; both *P* < 0.001), posterior tibial veins (left: 0.43 ± 0.52 vs. 0.24 ± 0.41, *P* = 0.032; right: 0.50 ± 0.55 vs. 0.21 ± 0.39, *P* = 0.003), and fibular veins (left: 0.67 ± 0.60 vs. 0.03 ± 0.40, *P* < 0.001; right: 0.55 ± 0.57 vs. 0.08 ± 0.38, *P* < 0.001). Additionally, the increase in Caprini score was significantly greater in the observation group than in the control group (2.45 ± 1.36 vs. 1.58 ± 1.21, *P* = 0.001). However, no statistically significant between-group difference was observed in Δ D-dimer levels (***P*** = 0.274).

**Table 5:** Between-group comparison of changes in outcome variables (Δ = T1−T0).

Variable	Control Group (*n* = 57)	Observation Group (*n* = 51)	*t*	*P*-value
Δ Caprini score	1.58 ± 1.21	2.45 ± 1.36	−3.52	0.001
Δ D-dimer (mg/L)	2.57 ± 2.31	2.13 ± 1.72	1.10	0.274
Δ Left popliteal vein velocity (cm/s)	0.07 ± 0.45	0.67 ± 0.58	−6.01	<0.001
Δ Right popliteal vein velocity (cm/s)	0.01 ± 0.48	0.65 ± 0.61	−5.78	<0.001
Δ Left posterior tibial vein velocity (cm/s)	0.24 ± 0.41	0.43 ± 0.52	−2.17	0.032
Δ Right posterior tibial vein velocity (cm/s)	0.21 ± 0.39	0.50 ± 0.55	−3.08	0.003
Δ Left fibular vein velocity (cm/s)	0.03 ± 0.40	0.67 ± 0.60	−6.42	<0.001
Δ Right fibular vein velocity (cm/s)	0.08 ± 0.38	0.55 ± 0.57	−4.89	<0.001

Values are presented as mean ± SD. Δ represents the difference between postoperative (T1) and preoperative (T0) measurements. Between-group comparisons were performed using the independent-samples *t*-test. The postoperative increase in Caprini score reflects accumulation of perioperative clinical risk factors rather than an intervention effect. Bold indicates statistically significant differences (*P* < 0.05).

### Multivariate linear regression analysis of hospital stay

Multivariate linear regression analysis was performed to identify independent factors associated with hospital stay (Table 6). The final regression model demonstrated good explanatory power (R^2^ = 0.62; adjusted R^2^ = 0.59). The results showed that electrophysiological therapy was independently associated with a shorter hospital stay (*β* = −0.98, 95% CI: −1.79 to −0.17, *P* = 0.018). In addition, greater improvement in venous blood flow velocity was independently associated with reduced hospitalization duration (*β* = −0.42, 95% CI: −0.72 to −0.12, *P* = 0.006). Conversely, age (*β* = 0.03, *P* = 0.006), operation time (*β* = 0.008, *P* = 0.011), and Caprini score (*β* = 0.18, *P* = 0.041) were positively associated with longer hospital stay. BMI was not significantly associated with hospitalization duration (*P* = 0.153).

**Table 6:** Multivariate linear regression analysis of factors associated with length of hospital stay.

Variable	*β*	SE	95% CI	*t*	*P*-value
Electrophysiological therapy	−0.98	0.41	−1.79 to −0.17	−2.39	**0** **.** **018**
Age	0.03	0.01	0.01 to 0.05	2.80	**0** **.** **006**
BMI	0.05	0.04	−0.02 to 0.12	1.44	0.153
Operation time	0.008	0.003	0.002 to 0.014	2.59	**0** **.** **011**
Caprini score	0.18	0.09	0.01 to 0.35	2.06	**0** **.** **041**
Δ Popliteal vein velocity (cm/s)	−0.42	0.15	−0.72 to −0.12	−2.80	**0** **.** **006**

*Β*, regression coefficient; SE, standard error; CI, confidence interval. Variables included in the multivariable model were selected based on clinical relevance and univariate analyses (*P* < 0.10). Model assumptions, including multicollinearity, linearity, homoscedasticity, and residual normality, were verified before interpretation. Model statistics: R^2^ = 0.62; adjusted R^2^ = 0.59. Bold indicates statistically significant associations (*P* < 0.05).

### Correlation between hemodynamic changes and clinical outcomes

Correlation analysis demonstrated that improvements in venous blood flow velocity were associated with better postoperative recovery (Table 7). Specifically, changes in popliteal vein velocity were moderately negatively correlated with hospital stay (r = −0.36, *P* = 0.001), while changes in fibular vein velocity showed a stronger negative correlation (r = −0.41, *P* < 0.001). In addition, changes in venous blood flow velocity exhibited weak negative correlations with D-dimer levels, particularly for fibular vein velocity (r = −0.25, *P* = 0.012). No statistically significant correlation was observed between posterior tibial vein velocity and D-dimer levels (*P* > 0.05).

**Table 7:** Correlation between changes in venous hemodynamics and clinical outcomes.

Variable	Hospital Stay (*r*)	*P* value	D-dimer (*r*)	*P*-value
Δ Popliteal vein velocity	−0.36	**0** **.** **001**	−0.21	**0** **.** **028**
Δ Posterior tibial vein velocity	−0.29	**0** **.** **004**	−0.18	0.061
Δ Fibular vein velocity	−0.41	**<0** **.** **001**	−0.25	**0** **.** **012**

Correlation coefficients were calculated using Pearson or Spearman correlation analyses, as appropriate. Negative correlation coefficients indicate that greater improvements in venous blood flow velocity were associated with shorter hospital stay or lower D-dimer levels. Bold indicates statistically significant correlations (*P* < 0.05).

### Adjusted analysis of outcomes

After adjusting for potential confounding factors, the observation group remained significantly higher in venous blood flow velocities and demonstrated a significantly shorter hospital stay compared with the control group (Table 8). The adjusted mean differences were significant for left popliteal vein velocity (mean difference=0.64, 95% CI: 0.25–1.03, *P* = 0.002) and right popliteal vein velocity (mean difference=0.61, 95% CI: 0.22–1.00, *P* = 0.003). Furthermore, the adjusted hospital stay remained significantly shorter in the observation group (mean difference=−0.99 days, 95% CI: −1.81 to −0.17, *P* = 0.016).

**Table 8:** Adjusted comparisons of primary outcomes after covariate adjustment.

Outcome	Adjusted Mean (Control)	Adjusted Mean (Observation)	Adjusted Mean Difference (95% CI)	*F*	*P*-value
Left popliteal vein velocity (cm/s)	4.31	4.95	0.64 (0.25 to 1.03)	10.84	**0** **.** **002**
Right popliteal vein velocity (cm/s)	4.40	5.01	0.61 (0.22 to 1.00)	9.96	**0** **.** **003**
Length of hospital stay (days)	7.91	6.92	−0.99 (−1.81 to −0.17)	6.01	**0** **.** **016**

Adjusted comparisons were performed using multivariable linear regression after controlling for age, body mass index, operation time, and Caprini score. Adjusted mean differences are presented with 95% confidence intervals. Bold indicates statistically significant differences (*P* < 0.05).

## Discussion

Patients undergoing laparoscopic nephrectomy are exposed to multiple perioperative factors that predispose them to venous thromboembolism (VTE), including surgical stress, pneumoperitoneum-induced venous stasis, prolonged operative time, and postoperative immobilization. These factors collectively contribute to a hypercoagulable state and increased thrombotic risk. If not effectively prevented, VTE may delay postoperative recovery, prolong hospitalization, and increase both individual and societal healthcare burdens. Therefore, optimizing perioperative VTE prevention strategies in this patient population is of considerable clinical importance (2). At present, commonly used VTE prophylactic strategies include basic measures, mechanical prevention, and pharmacological interventions. However, pharmacological prophylaxis may be limited by concerns regarding postoperative bleeding risk, while mechanical approaches such as graduated compression stockings and intermittent pneumatic compression devices often require prolonged use and may be associated with discomfort and poor compliance, potentially reducing their effectiveness (6, 8). These limitations highlight the need for alternative or adjunctive physical strategies that are both effective and well tolerated. Electrophysiological stimulation therapy, which integrates principles of traditional Chinese medicine and modern electrophysiology, has been increasingly explored as a non-invasive modality for improving venous circulation (7). Previous studies have demonstrated that electrical stimulation can enhance lower-extremity blood flow and may contribute to VTE prevention (9–13). However, evidence in patients undergoing laparoscopic nephrectomy remains limited. In the present study, electrophysiological therapy significantly improved lower-extremity venous hemodynamics compared with routine care alone, as reflected by increased blood flow velocities in the popliteal, posterior tibial, and fibular veins. Furthermore, change (Δ) analysis demonstrated that the magnitude of improvement in venous flow velocity was significantly greater in the observation group, particularly in the popliteal and fibular veins. These findings were further supported by correlation analysis, which revealed that improvements in venous blood flow velocity were moderately negatively correlated with hospital stay. In addition, multivariate regression analysis confirmed that electrophysiological therapy and improvements in venous hemodynamics were independent predictors of shorter hospitalization duration. Together, these findings suggest that electrophysiological therapy was associated with improved postoperative recovery, potentially through enhancement of venous return and reduction of venous stasis. However, because of the prospective non-randomized study design, these findings should be interpreted as associations rather than definitive evidence of causality.

The underlying mechanism of these effects can be explained by the classical Virchow's triad, which identifies venous stasis, hypercoagulability, and endothelial injury as the key contributors to thrombus formation (14). Electrophysiological stimulation induces rhythmic contraction and relaxation of lower limb muscles, mimicking the physiological muscle pump during ambulation. This promotes venous return, reduces venous stasis, and improves microcirculation, which is consistent with previous findings demonstrating improved blood flow velocities in distal lower-extremity veins following electrical stimulation (15). Additionally, low-frequency electrical stimulation may modulate neurovascular responses and promote vasodilation, further enhancing local blood and lymphatic circulation (7, 16). The absence of significant differences in femoral vein flow velocity between groups may be attributed to the larger vessel diameter and higher baseline flow, rendering it less sensitive to muscle pump effects. Treatment adherence was excellent, with all participants completing the planned intervention protocol. Only one participant experienced mild transient discomfort at the electrode placement site, which resolved spontaneously without treatment interruption or discontinuation. No serious intervention-related adverse events were observed, further supporting the feasibility, safety, and tolerability of electrophysiological stimulation as an adjunctive perioperative intervention.

In contrast, no significant between-group differences were observed in postoperative D-dimer levels or Caprini scores. Although the observation group demonstrated a significantly greater increase in Δ Caprini score, this finding most likely reflects the accumulation of postoperative clinical risk factors incorporated into the Caprini model rather than an actual increase in thrombotic burden. The Caprini score is a validated clinical risk stratification tool based primarily on patient characteristics and perioperative variables and is not designed to capture dynamic physiological improvements in venous hemodynamics induced by therapeutic interventions (17). Consequently, the greater postoperative increase in Caprini score should not be interpreted as evidence that electrophysiological stimulation increased thrombotic risk. Similarly, the absence of significant differences in D-dimer levels between groups may be explained by the relatively short postoperative observation period and the limited specificity of D-dimer in the immediate postoperative setting, where elevations frequently occur secondary to surgical trauma, tissue injury, and systemic inflammatory responses (18). Therefore, improvements in venous hemodynamics observed in the present study were not necessarily expected to be accompanied by measurable short-term changes in circulating D-dimer concentrations.

Nevertheless, the observation group demonstrated significantly shorter hospital stay and reduced bed rest duration compared with the control group, indicating improved postoperative recovery. These findings may be attributable to enhanced lower-limb circulation, reduced venous stasis, improved patient comfort, and earlier mobilization following electrophysiological stimulation (12). Improved comfort and early mobilization are important components of enhanced recovery after surgery (ERAS) protocols and may contribute to shorter hospitalization and more efficient utilization of healthcare resources (19, 20). Although no clinically confirmed VTE events occurred in either group during the study period, likely owing to the relatively small sample size, routine thromboprophylaxis, and short follow-up period, the observed improvements in venous hemodynamics and postoperative recovery provide supportive evidence that electrophysiological stimulation may enhance perioperative venous circulation. However, these findings should not be interpreted as demonstrating a reduction in clinical VTE incidence, and larger adequately powered studies with clinical VTE endpoints are required to determine whether these hemodynamic improvements translate into meaningful reductions in thromboembolic events.

The present findings should be interpreted in the context of the study design. Although electrophysiological stimulation was associated with favorable changes in venous hemodynamics and postoperative recovery, the absence of clinically confirmed VTE events precludes conclusions regarding its effectiveness in preventing thromboembolic complications. Instead, the results support the potential physiological benefits of electrophysiological stimulation as an adjunct to routine thromboprophylaxis. Future studies with larger sample sizes and clinically relevant endpoints are needed to determine whether these hemodynamic improvements ultimately translate into reductions in VTE incidence.

This study has several limitations. First, the prospective but non-randomized design with time-based allocation may have introduced selection bias and temporal confounding despite comparable baseline characteristics and statistical adjustment for clinically relevant covariates. Second, the sample size was relatively small and derived from a single center, which may limit the generalizability of the findings. Third, blinding of participants and treating clinicians was not feasible because of the nature of the intervention, although ultrasound outcome assessment was performed by experienced sonographers who were blinded to group allocation, thereby reducing the risk of detection bias. Fourth, the study primarily evaluated surrogate markers of venous hemodynamics and postoperative recovery rather than clinically confirmed VTE events, limiting direct assessment of clinical efficacy. Fifth, because this was an exploratory study, the analyses of secondary outcomes should be interpreted cautiously. Finally, the short follow-up period precluded evaluation of long-term outcomes. In addition, although the study received prospective institutional ethical approval before patient enrollment, it was not registered in a public clinical trial registry, which may limit methodological transparency. Despite these limitations, the present study has several strengths. It employed a prospective design, standardized perioperative management, blinded ultrasound assessment, objective evaluation of lower-extremity venous hemodynamics, and comprehensive assessment of both physiological and clinical recovery outcomes. Furthermore, multivariable regression analysis and adjusted analyses were performed to reduce the potential influence of measured confounding factors, thereby strengthening the robustness of the findings. Future multicenter randomized controlled trials with larger sample sizes, standardized perioperative management protocols, longer follow-up, and clinically confirmed VTE endpoints are warranted to validate these findings and determine whether the observed improvements in venous hemodynamics translate into clinically meaningful reductions in thromboembolic events. In addition, advanced vascular imaging, biomarker analyses, and mechanistic investigations may further elucidate the physiological effects of electrophysiological stimulation on venous hemodynamics, endothelial function, and coagulation pathways.

## Conclusion

Electrophysiological stimulation therapy was associated with improved lower-extremity venous hemodynamics in patients undergoing laparoscopic nephrectomy, as evidenced by increased blood flow velocity in distal lower-limb veins. In addition, this intervention was associated with shorter bed rest duration and reduced length of hospital stay, suggesting an association with enhanced postoperative recovery. No clinically confirmed VTE events were observed during the study period; therefore, the present findings do not demonstrate a reduction in VTE incidence. Nevertheless, the observed improvements in venous hemodynamics and recovery parameters suggest that electrophysiological stimulation may serve as a useful adjunct to conventional thromboprophylaxis in the perioperative management of patients undergoing laparoscopic nephrectomy. As a non-invasive, well-tolerated, and easily applicable modality, electrophysiological stimulation represents a feasible adjunctive strategy for enhancing perioperative venous circulation. However, given the prospective non-randomized design, single-center setting, modest sample size, and reliance on surrogate hemodynamic outcomes rather than clinical VTE endpoints, further large-scale, multicenter randomized controlled trials are required to confirm these findings and determine whether the observed improvements in venous hemodynamics translate into clinically meaningful reductions in VTE incidence.

## Data Availability

The original contributions presented in the study are included in the article/Supplementary Material, further inquiries can be directed to the corresponding authors.
